# Metal-on-metal hip resurfacing in patients younger than 50 years: a retrospective analysis: 1285 cases, 12-year survivorship

**DOI:** 10.1186/s13018-017-0579-y

**Published:** 2017-06-02

**Authors:** Melissa D. Gaillard, Thomas P. Gross

**Affiliations:** Midlands Orthopaedics & Neurosurgery, 1910 Blanding Street, Columbia, SC 29201 USA

**Keywords:** Hip resurfacing, Metal-on-metal, Younger patients, Hip arthroplasty

## Abstract

**Background:**

The Nordic registry reports patients under 50 years old with total hip replacements realize only 83% 10-year implant survivorship. These results do not meet the 95% 10-year survivorship guideline posed by the UK’s National Institute for Health and Care Excellence (NICE) in 2014.

**Methods:**

The purpose of this study is threefold: First, we evaluate if metal-on-metal hip resurfacing arthroplasty meets these high standards in younger patients. Next, we compare outcomes between age groups to determine if younger patients are at higher risk for revision or complication. Lastly, we assess how outcomes between sexes changed over time. From January 2001 to August 2013, a single surgeon performed 1285 metal-on-metal hip resurfacings in patients younger than 50 years old. We compared these to an older cohort matched by sex and BMI.

**Results:**

Kaplan-Meier implant survivorship was 96.5% at 10 years and 96.3% at 12 years; this did not differ from implant survivorship for older patients. Implant survivorship at 12 years was 98 and 93% for younger men and women, respectively; survivorship for women improved from 93 to 97% by using exclusively Biomet implants. There were four (0.3%) adverse wear-related failures, with no instances of wear or problematic ion levels since 2009. Activity scores improved from 5.4 ± 2.3 preoperatively to 7.6 ± 1.9 postoperatively (*p* < 0.0001), with 43% of patients reporting a UCLA activity score of 9 or 10.

**Conclusions:**

Hip resurfacing exceeds the stricter 2014 NICE survivorship criteria independently in men and women even when performed on patients under 50 years old.

## Background

Total hip arthroplasty (THA) is durable in elderly populations [1] but does not meet functional demands or durability requirements for younger patients [2–5]. In 2014, the UK’s National Institute for Health and Care Excellence (NICE) raised their benchmark criteria for hip implants from 90 to 95% 10-year survivorship. The Orthopaedic Data Evaluation Panel listed only 32 THA femoral stems which met this new, stricter benchmark [6], with potentially far fewer meeting NICE criteria in younger patients.

Sir John Charnley warned against performing THA in younger patients, citing that the procedure was not robust enough [7]; as an increasing number of younger patients demanded better, longer-lasting solutions [8], hip resurfacing arthroplasty (HRA) emerged as an alternative, bone-preserving option. It is well known THA implants display markedly lower survivorship in patients younger than 50 years old [2–4]. In the Scandinavian registry, 10-year implant survivorship for these patients was only 83% [5]. Considering underestimation of failure in the registry, 10-year survivorship could be lower. In a literature review by De Kam on THA in patients under 50 [9], only 15 of 37 papers met the outdated NICE criteria, and of these, only two studies met the new standard.

Experts attribute reduced implant survivorship in younger patients to more complex procedures and naturally higher activity levels [3, 10, 11]. The most common diseases of the younger hip include osteonecrosis, dysplasia, Legg-Perthes disease, and post-traumatic arthritis, all of which carry a worse prognosis [12]. Aside from having higher expectations, younger patients often require eventual revision; in an analysis of over 109 studies on patients under 50, only 37 had a mean survivorship greater than 10 years [9]. These combined risks make THA challenging in patients under 50.

McMinn et al. [13] and Amstutz [14] introduced metal-on-metal (MOM) HRA as a bone-preserving, temporizing measure to delay disease progression in younger patients, but HRA far surpassed these modest, early goals. Recent advancements in MOM bearing design have improved durability and lowered wear rate, with many studies reporting 93% implant survivorship for patients in their 40s [15–17]. Furthermore, several studies suggest that gait characteristics are more nearly normal in those receiving HRA versus THA [18, 19], appealing especially to younger patients.

HRA, compared to THA, allows for a more natural reconstruction of the hip and endows biomechanics more closely resembling a normal, healthy joint. The naturally stable bearing size and femoral offsets are preserved, leading to superior hip stability [20, 21]. The lack of a large stem has resolved issues with thigh pain [10, 22]. Gait lab studies demonstrate THA patients do not fully load the operative leg and take smaller strides than HRA patients [18]. These combined advantages of HRA allow more nearly normal function in younger patients who often still desire to participate in high range-of-motion (ROM) activities and impact sports. The available scientific studies amply confirm that HRA is a more functional arthroplasty than THA [23–25].

Despite many HRA studies with excellent outcomes, registry results have been mixed. While the Australian registry [26] confirms 10-year HRA implant survivorship surpasses that of THA in men under 60, the reverse is true for women and older men. In the UK’s National Joint Registry, Smith et al. demonstrated HRA only outlives THA in men with larger implant sizes [27], but the study included inexperienced surgeons performing an average of only 2.6 HRA cases a year. Publications from inexperienced surgeons with weak results, high failure rates from poorly designed implants, and excessive publicity on adverse wear-related failures (AWRF) from a small number of outlier centers [28] have called into question the value of HRA. However, experienced surgeons, including the present senior author (TPG), have routinely surpassed HRA and THA outcomes in arthroplasty registers [5, 27, 29]. Due to the mixed available results, the scarcity of published outcomes on younger patients, and the poor viability of THA in younger patients, we aim to establish a successful example for HRA implant survivorship in patients under 50 years old.

In 2001, the senior author began performing HRA on the basis that bone preservation in younger patients is paramount. We present the results of 1285 HRA procedures performed on patients under 50 years old and compare these data with a demographically similar, older cohort to evaluate several hypotheses:

1. MOM HRA meets the 2014 NICE criteria in our patients under 50 years old.
2. There is no difference in HRA implant survivorship due to age.
3. Outcome disparity between sexes of our younger cohort has improved.

## Methods

### Patients and follow-up

We used OrthoVault (Midlands Orthopaedics & Neurosurgery, Columbia, SC), our database of over 4200 HRA procedures, to retrospectively identify 1285 consecutive cases from January 2001 to August 2013 in 1062 patients under 50 years old at the time of surgery as our group 1 study cohort. Ages for group 1 ranged from 11 to 49 years. From the same date range, we identified 1984 HRA devices in 1614 patients as the older control group (group 2); ages among the group 2 cohort ranged from 50 to 78 years. These patients received surgery at the age of 50 years or older. All patients had a minimum of 2 years’ follow-up. Between January 2001 and January 2005, the primary surgeon performed 372 HRA procedures in 329 patients using the hybrid Corin Cormet 2000 resurfacing system. Subsequently, from January 2005 to March 2007, the primary surgeon performed 739 HRA procedures in 652 patients using the hybrid Biomet ReCap™-Magnum™ implant system. Lastly, we shifted to our current uncemented method, and from March 2007 to August 2013, 1803 patients received 2158 fully porous-coated Biomet ReCap™-Magnum™ resurfacings.

Table 1 presents demographic information for both cohorts. The average age at the time of surgery for group 1 was 44 ± 6 years and for group 2 was 57 ± 4 years. Bone density was greater for younger patients (see “Statistical methods”). Sex distribution and BMI were not statistically different between the two groups. Group 1 presented a higher percentage of patients with more complex diagnoses, which are those that typically result in worse outcomes.

**Table 1:** Group demographics for patients under and over 50 years old

	Group 1, <50 (*N* = 1285)	Group 2, ≥50 (*N* = 1984)	*p* value
Sex (no. of hips)			
Male	951 (74%)	1426 (72%)	0.1802
Female	334 (26%)	558 (28%)	
Deceased^#^	10 (0.8%)	29 (1.5%)	0.0784
F/U mean years	3.4 ± 2.98	2.8 ± 2.59	<0.0001*
Lost to F/U	17 (1.3%)	23 (1.2%)	0.6745
Case date range	1/2001–8/2013		–
Age (years)	44 ± 6.02	57 ± 4.23	<0.0001*
BMI	28 ± 4.92	28 ± 4.56	0.0750
*T*-score	0.26 ± 1.36	−0.14 ± 1.18	<0.0001*
Uncemented fixation (no. of hips)	776 (60%)	1380 (70%)	<0.0001*
10-year survivorship (no. of hips)	1234 (96%)	1924 (97%)	0.1443
Diagnosis (no. of hips)			
Dysplasia	149 (12%)	214 (11%)	0.4715
Osteoarthritis (OA)	866 (67%)	1589 (80%)	<0.0001*
Osteonecrosis (ON)	107 (8.3%)	63 (3.2%)	<0.0001*
Rheumatoid arthritis (RA)	9 (0.7%)	2 (0.1%)	0.0039*
Post-trauma	40 (3.1%)	20 (1.0%)	<0.0001*
Legg-Calve-Perthes disease (LCP)	32 (2.5%)	7 (0.4%)	<0.0001*
Slipped capital femoral epiphysis (SCFE)	12 (0.9%)	9 (0.5%)	0.0930
Other	22 (1.7%)	21 (1.1%)	0.1096
Implants (no. of hips)			
Corin Cormet 2000	187 (14%)	185 (9.3%)	<0.0001*
Biomet ReCap™-Magnum™ hybrid	330 (26%)	409 (21%)	0.0007*
Biomet ReCap™-Magnum™ uncemented	768 (60%)	1390 (70%)	<0.0001*
ASA score	1.6 ± 0.57	1.7 ± 0.58	<0.0001*
Femoral component <48 mm (no. of hips)	199/973 (20%)	334/1641 (20%)	0.9522
Femoral component size	50.0 ± 3.92	50.2 ± 3.53	0.1793

Statistically significant *p* values are italicized and denoted by an asterisk (*)

^#^indicates deaths unrelated to the patients' hip arthroplasties

This study is a level II, retrospective review of prospectively collected data. Approval for this study and manuscript was granted by the Institutional Review Board of Providence Health in Columbia, SC.

### Implant systems

The primary surgeon (TPG) performed HRAs using three unique implant systems in a consecutive fashion. We began using the hybrid Corin Cormet 2000 (Corin, Cirencester, UK) implant system in March 2001 as part of a multicenter US Food and Drug Administration clinical trial. This device was fully approved in 2007 but is no longer sold. We partnered with Biomet (now Zimmer Biomet) to develop the hybrid ReCap™-Magnum™ (Biomet, Warsaw, IN), which we began using in 2005. We further collaborated with Biomet to develop the fully porous-coated ReCap™-Magnum™, which became available in March 2007; we have used this exclusively for all resurfacing cases after January 2008. In the USA, employing the ReCap™-Magnum™ system for HRA is considered off-label use. We published comprehensive metallurgy and design details for all implant systems previously [30, 31].

### Procedure

The primary surgeon (TPG) performed all HRA operations through the posterior approach as described previously [32]. We have taken normalized to standing intraoperative radiographs since 2009 to confirm the acetabular component position meets our relative acetabular inclination limit (RAIL) guideline [33]. Table 2 presents a summary of surgical information.

**Table 2:** Surgical summary for two groups

Variable	Group 1	Group 2	*p* value
Length of incision (in.)	4.4 ± 1.44	4.3 ± 0.77	0.0100*
Operation time (min)	106 ± 19.4	102 ± 28.8	<0.0001*
Estimated blood loss (mL)	208 ± 171	183 ± 137	<0.0001*
Hospital stay (day)	2.1 ± 1.11	2.1 ± 5.17	1.0000
Transfusion received (no. of cases)	0 (0.0%)	2 (0.1%)	0.2543
Transfusion volume (cm^3^)	**–**	375 ± 0	**–**
Outpatient (no. of cases)	10 (0.8%)	21 (1.1%)	0.4179

Statistically significant *p* values are italicized and denoted by an asterisk (*)

### Postoperative protocol

Patients progress to weight-bearing as tolerated unless they present notably low preoperative bone density. Most patients use crutches for 2 weeks and a cane for 2 weeks thereafter. We require no formal physical therapy following hospital discharge. Patients may progress to moderate aerobic exercise at 6 weeks and unlimited activity at 6 months after surgery. The establishment of a multimodal pain management protocol and comprehensive blood management protocol has accelerated patient recovery and eliminated the need for transfusion, allowing many patients to receive HRA as an outpatient procedure since 2012.

### Metal ion testing

The OrthoVault database facilitates collection of metal ion test results, which we routinely requested from all patients at 2 years postoperatively since 2007; we also requested metal ion results from all patients operated on prior to this time at least once. Metal ion levels are useful indicators of potential failure from excessive implant wear [34] even before the onset of symptoms. We converted serum and plasma test results for cobalt (Co) and chromium (Cr) to whole blood ion level values using Smolders’ method [35, 36] and subsequently used whole blood values for all comparisons. Based on previous research [33–35], we define five ion level categories (Table 3): normal, optimal, acceptable, problematic, and potentially toxic.

**Table 3:** Whole blood metal ion reference table

	Normal^a^	Optimal^b^	Acceptable^c^	Problematic^c^	Potentially toxic^b^
Unilateral					
Co (μg/L)	<1.5	<4.0	4–10	10–20	>20
Cr (μg/L)	<1.5	<4.6	4.6–10	10–20	>20
Bilateral					
Co (μg/L)	<1.5	<5.0	5–10	10–20	>20
Cr (μg/L)	<1.5	<7.4	7.4–10	10–20	>20

^a^Laboratory normal for patients without metal bearings

^b^According to DeSmet/Van der Straeten [34, 35]

^c^According to our previous analysis

### Clinical and radiographic analysis

We request patients return for an office visit or to complete a remote follow-up package at 6 weeks, 1 and 2 years, and every other year thereafter. Each follow-up comprises a clinical questionnaire, radiographic analysis, and a physical examination testing ROM and strength. Physical examinations are no longer necessary after the 1-year postoperative visit for patients completing remote follow-up. OrthoVault supported the collection of demographic, clinical, and radiographic data for all patients.

We use clinical questionnaires to collect information for calculating the following scores: Harris hip score (HHS) [37], University of California, Los Angeles (UCLA) activity score [38], and visual analog scale (VAS) pain scores [39]. We use the HHS for quantitative measurement of overall clinical outcome, based on function and ROM. UCLA activity scores measure patient activity level on a scale of 1 to 10, for which 10 represents regular participation in impact sports. VAS pain scores provide a simple indication of overall pain on normal and worst days based on a scale of 0, or no pain, to 10, or maximum, debilitating pain.

**Fig. 1:**
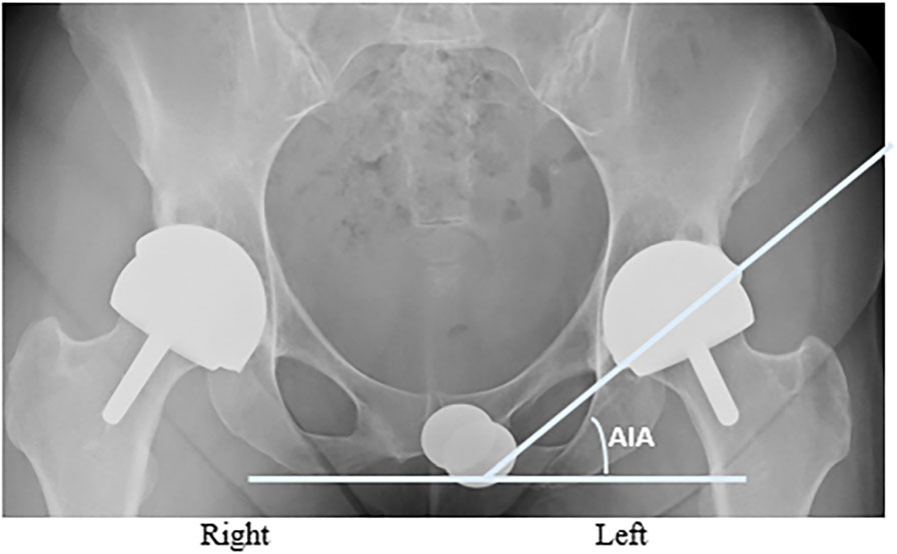
Pelvic radiograph acetabular inclination angle measurement lines. Anterior-posterior pelvis radiograph taken 5 years after a hybrid metal-on-metal Corin hip resurfacing on the right hip and 2 years after a hybrid metal-on-metal Biomet ReCap™ hip resurfacing on the left hip. Better AIA is noted in the most recent HRA on the patient’s left side

Radiographs are obtained at every follow-up; these x-rays are analyzed for component position, shifting, and radiolucencies. We determine the acetabular inclination angle (AIA) by measuring the angle formed between a horizontal reference line running across the face of the inferior pubic rami and a measurement line running across the face of the acetabular component on the patient’s standing anterior-posterior x-ray (Fig. 1). All measurements were performed using OrthoVault and InteleViewer® (InteleRAD, Chicago, IL, USA).

### Statistical methods

Statistical analyses were performed using Microsoft® Excel (Microsoft, Redmond, WA, USA) and SAS® (SAS Institute Incorporated, Cary, NC, USA). All tests used a significance level of *α* = 0.05. Paired, two-tailed Student’s *t* tests were used to find significant differences between numeric results. Two-sample proportion *Z*-tests were performed to compare percentages. Kaplan-Meier (KM) implant survivorship curves were plotted using XLSTAT® (Addinsoft, New York, NY, USA), and log-rank and Wilcoxon tests were performed to determine significant difference in implant survivorship between groups.

## Results

### Survivorship

**Fig. 2:**
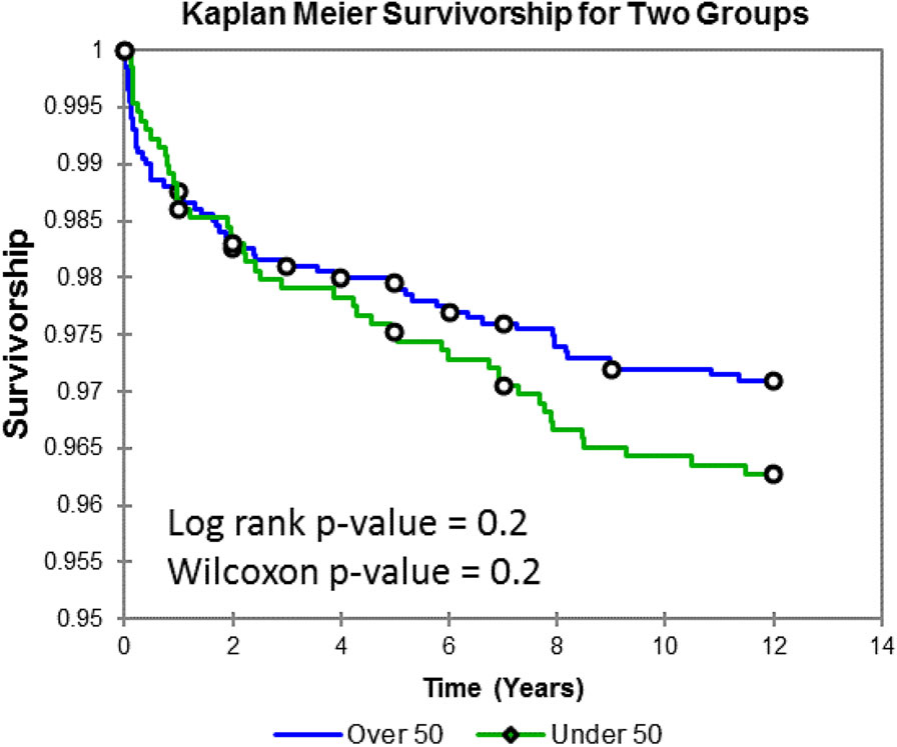
Kaplan-Meier implant survivorship curves for two study cohorts. *Open circles* represent deaths unrelated to the patients’ hip arthroplasties

**Fig. 3:**
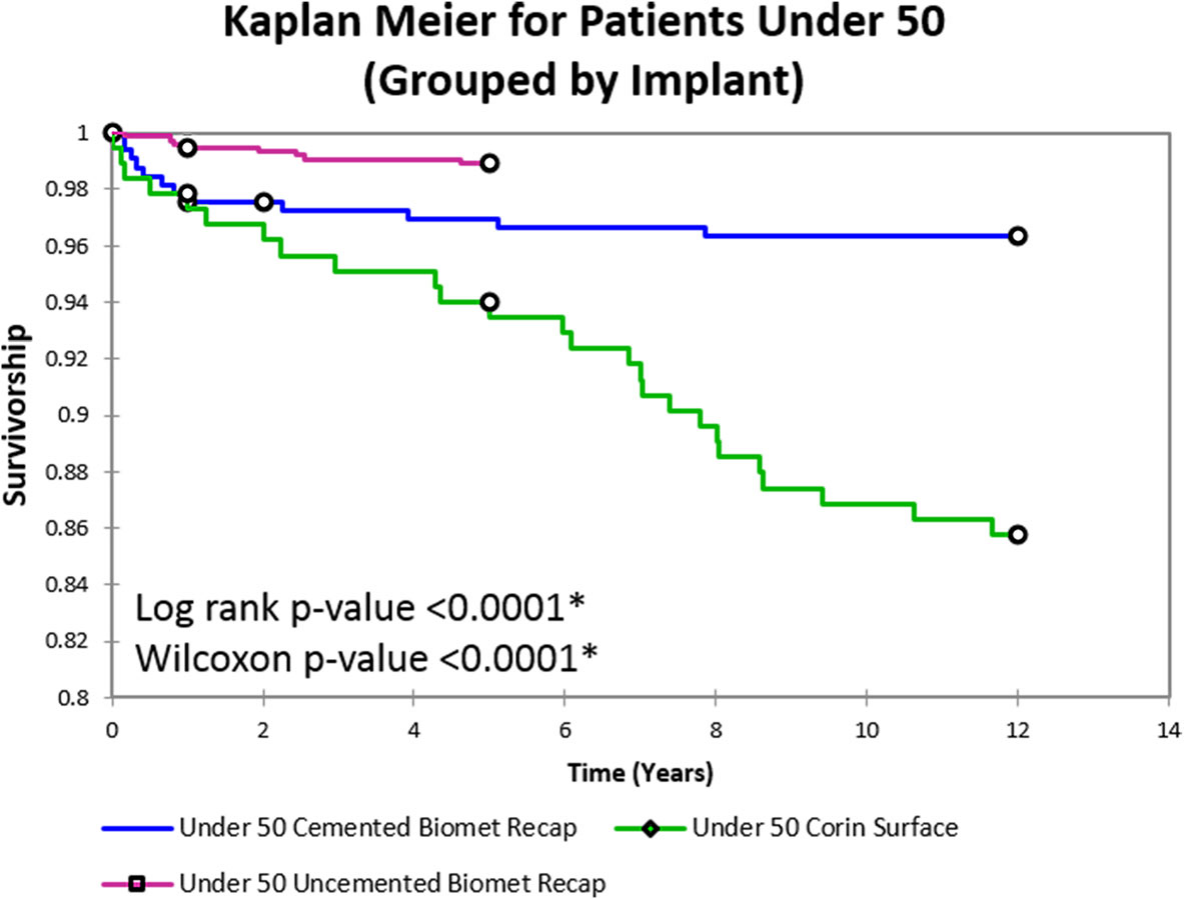
Kaplan-Meier implant survivorship curves for patients under 50 grouped by implant. *Open circles* represent deaths unrelated to the patients’ hip arthroplasties. *Asterisk* represents statistical significance

KM implant survivorship (Fig. 2) at 10 years was 96.5% and at 12 years was 96.3% for patients under 50. Overall survivorship also improved with each successive implant type (Fig. 3); for both age groups, the uncemented ReCap™-Magnum™ system exhibited significantly better survivorship than all other implants at 8 years (group 1 *p* < 0.0001, group 2 *p* = 0.001). Survivorship did not vary by age for any implant (log-rank *p* = 0.199 and Wilcoxon *p* = 0.206).

**Fig. 4:**
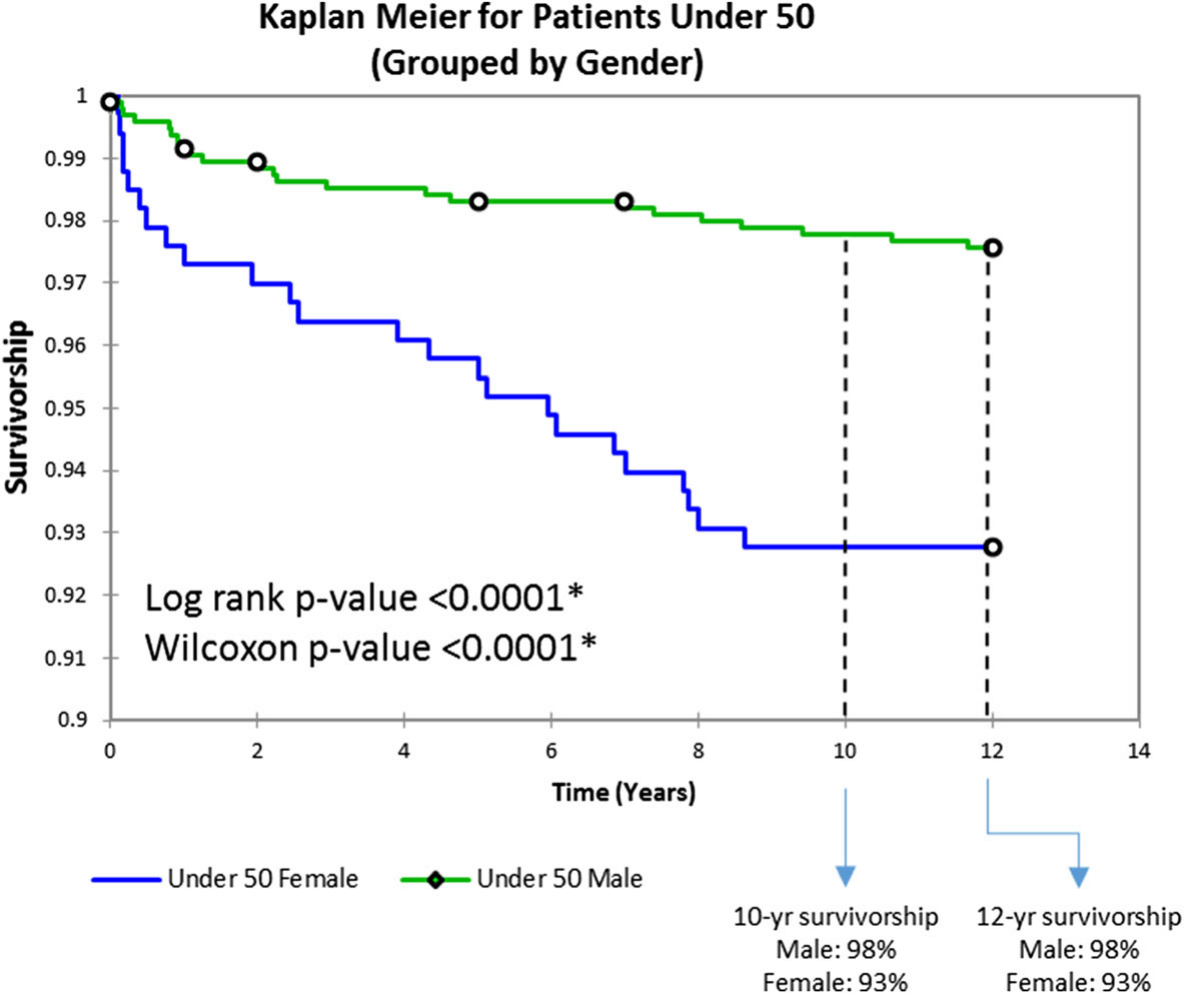
Kaplan-Meier implant survivorship curves for under 50 grouped by sex. *Open circles* represent deaths unrelated to the patients’ hip arthroplasties. *Asterisk* represents statistical significance

**Fig. 5:**
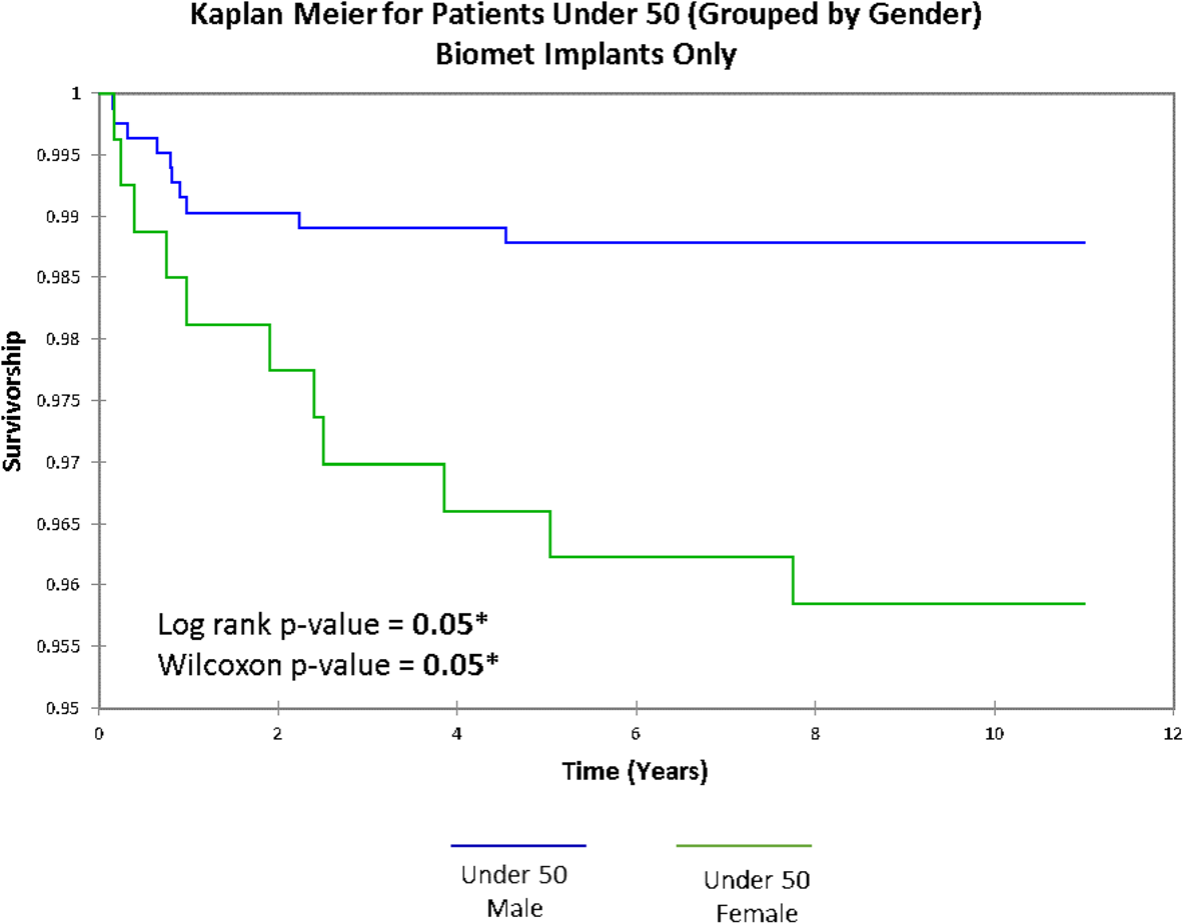
Kaplan-Meier implant survivorship curves by sex for Biomet implants *only. Asterisk* represents statistical significance

Survival rates varied by sex (Fig. 4), with males displaying significantly greater implant survivorship at 12 years than females in both group 1 (98 vs. 93%, respectively, log-rank and Wilcoxon *p* < 0.0001) and group 2 (99 vs. 95%, respectively, log-rank and Wilcoxon *p* < 0.0001). Sex disparity decreased with each successive implant type. Disparity in male-female results was minimal in cases with the uncemented Biomet ReCap™-Magnum™, with an 8-year failure rate of 99.5 and 97.0% for group 1 males and females, respectively (log-rank and Wilcoxon *p* = 0.01) (Fig. 5).

### Failures

Table 4 details modes of failure and indicates for each failure type whether there is or is not significant difference. The only statistically significant difference in occurrence of any failure mode was that of recurrent instability, with which was greater in group 1 (0.2% in group 1 and 0.0% in group 2, *p* = 0.03). AWRF was rare (0.3% in group 1 and 0.4% in group 2, *p* = 0.84) with no instances of wear in cases performed after July 2009; there was no significant difference in AWRF between age groups (*p* = 0.84). One of four total cases of unexplained pain occurred in group 1 (*p* = 0.55). This female patient received revision surgery 1 year after her original operation. Preceding revision, whole blood Co and Cr ion levels were 10.8 and 4.5 μg/L, respectively. Her CT scan prior to revision revealed a small, 3-cm fluid collection anteriorly. While this evidence suggests mild AWRF, implants were found well fixed at the time of surgery, with minimal osteolysis of the acetabulum and femur. All symptoms resolved by 3 months post-revision, and the patient scored a 100 HHS on their most recent 2-year follow-up.

**Table 4:** Failures for two study groups

Type	Group 1	Group 2	*p* value
No. of cases	1285	1984	–
1) Acetabular failures			
Adverse wear	4 (0.3%)	7 (0.4%)	0.8415
Loose acetabular component	6 (0.5%)	4 (0.2%)	0.1802
Failure of acetabular ingrowth	10 (0.8%)	9 (0.5%)	0.2340
Acetabular component shift	0 (0%)	1 (0.1%)	0.4237
2) Femoral failures			
Early femoral neck fracture	5 (0.4%)	15 (0.8%)	0.1902
Loose femoral component	12 (1.0%)	9 (0.5%)	0.0930
Femoral head collapse	2 (0.2%)	4 (0.2%)	0.7642
3) Other failures			
Unexplained pain	1 (0.1%)	3 (0.2%)	0.5552
Late infection	2 (0.2%)	0 (0%)	0.0784
Early infection	1 (0.1%)	1 (0.1%)	0.7566
Late fracture	2 (0.2%)	1 (0.1%)	0.3320
Recurrent instability	3 (0.2%)	0 (0%)	0.0316*
Psoas tendonitis	1 (0.1%)	0 (0%)	0.2150
Other failures^a^	0 (0%)	4 (0.2%)	0.1074
Total failures	48 (3.7%)	58 (2.9%)	0.2005

Statistically significant *p* values are italicized and denoted by an asterisk (*)

^a^Other causes include diarrhea, UTI, urinary retention, squeaking implant, frostbite, and other uncommon causes not built into the database

### Complications and reoperations

Table 5 lists complications, and Table 6 details reoperations. Group 2 patients were more likely to experience acetabular component shift not resulting in reoperation or revision than group 1 (0.9 vs. 0.2%, respectively, *p* = 0.007). All 21 recognized cases of acetabular shift occurred before 6 weeks and stabilized. All shifted components, with a single exception, became more horizontal than their initial position, and all patients presented optimal metal ion levels.

**Table 5:** Complications for two study groups

Type	Group 1	Group 2	*p* value
No. of cases	1285	1984	–
Complications			
1) Acetabular complications			
Loose acetabular component	1 (0.1%)	0 (0%)	0.2150
Acetabular component shift	2 (0.2%)	18 (0.9%)	0.0071*
2) Femoral complications			
Loose femoral component	1 (0.1%)	1 (0.1%)	0.7566
Femoral component shift	0 (0%)	1 (0.1%)	0.4237
3) Other complications			
Psoas tendonitis	1 (0.1%)	0 (0%)	0.2150
Sciatic nerve palsy	3 (0.2%)	2 (0.1%)	0.3421
Hip dislocation	4 (0.3%)	12 (0.6%)	0.2420
Late fracture	2 (0.2%)	2 (0.1%)	0.6599
Pulmonary embolus	3 (0.2%)	2 (0.1%)	0.3421
Spinal headache	2 (0.2%)	1 (0.1%)	0.3320
Embolic stroke	1 (0.1%)	2 (0.1%)	0.8337
Unexplained pain	0 (0%)	3 (0.2%)	0.1645
Psoas hematoma	1 (0.1%)	0 (0%)	0.2150
Abductor tear	0 (0%)	3 (0.2%)	0.1645
Deep vein thrombosis	2 (0.2%)	5 (0.3%)	0.5619
Other complications^a^	1 (0.1%)	9 (0.5%)	0.0574

Statistically significant *p* values are italicized and denoted by an asterisk (*)

^a^Other complications include diarrhea, spinal headache, urinary retention, squeaking implant, and other uncommon causes not built into the database

**Table 6:** Reoperations for two study groups

Type	Group 1	Group 2	*p* value
No. of cases	1285	1984	–
Reoperations			
Femoral neck fracture	0 (0%)	1 (0.1%)	0.4237
Early fracture	0 (0%)	1 (0.1%)	0.4237
Late fracture	2 (0.2%)	2 (0.1%)	0.6599
Fascial healing defect	1 (0.1%)	0 (0%)	0.2150
Psoas tendonitis	1 (0.1%)	1 (0.1%)	0.7566
Late infection	0 (0%)	2 (0.1%)	0.2543
Early infection	2 (0.2%)	4 (0.2%)	0.7642
Wound dehiscence	2 (0.2%)	2 (0.1%)	0.6599
Other causes^a^	2 (0.2%)	0 (0%)	0.0784

^a^Other causes include suture reaction, frostbite, and other uncommon causes not built into the database

The overall rate of instability not resulting in revision surgery was 0.3% in group 1 and 0.6% in group 2 (*p* = 0.24). These were treated nonoperatively, and all patients scored a HHS ≥ 92 by 1-year post-revision and presented acceptable blood metal ion levels after surgery.

### Ion data and adverse wear-related failure

Approximately 65% of patients from both groups complied with our request for metal ion levels (Table 7). Group 2 unilateral patients expressed slightly higher mean Cr levels (*p* = 0.05), although the difference in mean Cr levels was nonsignificant between the two bilateral cohorts (*p* = 0.28). Average Co ion levels were not statistically different between age groups for either unilateral (*p* = 1.0) or bilateral (*p* = 0.26) patients. Cobalt levels in 825 group 1 patients were optimal in 99% of unilateral cases and 97% of bilateral cases, with no levels greater than 10 μg/L, excluding revised cases. All patients presenting with AWRF in group 1 had ion levels ≥14 μg/L and were revised successfully. Four patients from group 1, and seven from group 2, have developed AWRF to date (*p* = 0.84) (Table 2). The most recent case that resulted in ion levels greater than 10 μg/L was a case from June 2009; this was the last case to require revision for AWRF. Seven years have elapsed, and in this study, 1530 cases have been performed since that time.

**Table 7:** Whole blood metal ion results

Under 50 case study								
Variables	Group 1 (under 50)			Group 2 (over 50)			*p* values between group 1 and group 2	
	Unilateral (*N* = 494)	Bilateral (*N* = 331)	*p* value	Unilateral (*N* = 836)	Bilateral (*N* = 559)	*p* value	Unilateral 1 vs. 2	Bilateral 1 vs. 2
Co (μg/L)	1.1 ± 0.83	1.8 ± 1.25	<0.0001*	1.1 ± 0.93	1.9 ± 1.31	<0.0001*	1.000	0.2633
Cr (μg/L)	0.9 ± 0.84	1.6 ± 1.32	<0.0001*	1.0 ± 0.93	1.5 ± 1.32	<0.0001*	0.0498*	0.2750
Follow-up date (years)	4.4 ± 2.58	4.9 ± 2.75	0.0080*	3.9 ± 2.38	4.4 ± 2.55	0.0002*	0.0003*	0.0062*
No. (%) of patients tested	825 (64%)		–	1359 (70%)		–	0.0108*	
No. (%) of levels converted	117 (24%)	72 (22%)	0.5157	191 (23%)	138 (25%)	0.4295	0.7279	0.3173
Normal, no. (%)	393 (80%)	169 (51%)	<0.0001*	657 (79%)	234 (42%)	<0.0001*	0.6745	0.0078*
Optimal, no. (%)	488 (99%)	320 (97%)	0.0366*	815 (97%)	533 (95%)	0.0300*	0.1052	0.3371
Acceptable, no. (%)	6 (1.2%)	11 (3.3%)	0.0366*	21 (2.5%)	26 (4.7%)	0.0300*	0.1052	0.3371
Problematic, no. (%)	0 (0%)	0 (0%)	1.000	0 (0%)	0 (0%)	1.000	1.000	1.000
Potentially toxic, no. (%)	0 (0%)	0 (0%)	1.000	0 (0%)	0 (0%)	1.000	1.000	1.000

Statistically significant *p* values are italicized and denoted by an asterisk (*)

### Clinical data

Clinical outcomes for unrevised cases are presented in Table 8. Postoperative average HHS (97 ± 7) was similar for the two groups (*p* = 1.0). Postoperative UCLA activity scores were significantly higher for group 1 (*p* = 0.003). VAS pain scores on regular days were statistically equivalent between the two groups (*p* = 1.0). VAS pain scores on worst days were lower for group 2 (*p* < 0.0001).

**Table 8:** Clinical follow-up information for two study groups

Variable	Group 1—under 50	Group 2—over 50	*p* value
Preoperative			
HHS	58 ± 6.47	49 ± 7.56	<0.0001*
Postoperative			
HHS	97 ± 6.80	97 ± 6.60	1.000
UCLA activity score	7.6 ± 1.91	7.4 ± 1.90	0.0033*
High-impact UCLA, no. of cases (%)	230/539 (43%)	377/1031 (37%)	0.0183*
VAS pain: regular	0.2 ± 0.80	0.2 ± 0.74	1.000
VAS pain: worse	1.5 ± 2.07	1.1 ± 1.78	<0.0001*
Combined ROM	258 ± 46.1	264 ± 41.9	<0.0001*
Radiographic data			
AIA	39.6 ± 8.14	39.6 ± 8.15	1.000
Under RAIL, no. of hips (%)	1138/1238 (92%)	1807/1918 (94%)	0.0366*
Radiolucency, no. of hips (%)	0 (0.0%)	3 (0.2%)	0.1645
Osteolysis, no. of hips (%)	0 (0.0%)	0 (0.0%)	1.000

Statistically significant *p* values are italicized and denoted by an asterisk (*)

### Radiographic data

Radiographic data for unrevised cases are presented in Table 8. The mean AIA was 40° for both groups (*p* = 1.0). Fewer group 1 cases met our RAIL criteria for proper component position (92 vs. 94%, *p* = 0.04). There were no cases exhibiting lysis (*p* = 1.0), while three cases in the older cohort displayed limited partial radiolucency (*p* = 0.16).

### Surgical data

Length of incision, operation time, and estimated blood loss were greater in group 1 (*p* = 0.01, *p* < 0.0001, and *p* < 0.0001, respectively). However, no transfusions were required, and hospital stay did not differ between the two age groups (*p* = 1.0).

## Discussion

These data evince the validity of all of our original hypotheses. In the largest single-center report of hip arthroplasty yet published for patients under 50 years old, we have demonstrated that MOM hip resurfacing exceeds the stricter 2014 NICE benchmark of 95% 10-year implant survivorship. We achieved 96.5% at 10 years and 96.3% at 12 years in this unselected, consecutive group of 1285 patients under 50 years of age.

Similar to other studies [40–43], we confirmed that hip resurfacing has better implant survivorship in men (*p* < 0.0001) (Fig. 4). However, disparity in results by sex was reduced when we considered only the latest uncemented Biomet cohort, with 8-year survivorship at 99.5% in males and 97.0% in females; per the Orthopaedic Data Evaluation Panel [6], both groups are independently on track to exceed the 2014 NICE criteria. These data show that HRA in women now achieves similar outcomes as in men and that the sex disparity has nearly been resolved.

From the current data, it becomes evident that as hip resurfacing matures, as the scientific body of evidence in resurfacing grows, as failure modes are studied and solutions are found, as implant designs improve, and as surgeon experience grows, clinical outcomes and implant survivorship improve. It is impossible to determine the exact interplay of these complex factors without further studies, but Fig. 2 undeniably shows dramatic improvement in overall implant survivorship over the 12-year period that encompasses this study. In this short time, 8-year implant survivorship increased from 88 to 99% (log-rank *p* < 0.0001, Wilcoxon *p* < 0.0001). While implant survivorship in THA is generally much lower in younger patients, this does not hold true for hip resurfacing at our center. KM implant survivorship curves comparing our younger and older patient cohorts (Fig. 2) show no statistical difference in failure rates. These KM data show hip resurfacing implants in younger, active patients are at no higher risk of failure.

It is noteworthy that we have achieved 2014 NICE survivorship in our younger, high-risk population, as this group typically presents poor outcomes in THA [2, 9]. Our study produced better outcomes than most THA reports and compare favorably to other smaller, youth-centered resurfacing studies, such as those by Sayeed et al., Haddad et al., and Krantz et al. (Table 9) [11, 23, 44]. Similar survivorship in patients under 50 has been achieved with the Birmingham hip resurfacing, with one report of 100% 10-year survivorship in 20 hips [23] and another report of 96% 10-year survivorship in 447 hips [45]. We are aware of two recent series of uncemented THAs that have achieved similar success, including a report by Facek et al. [46] of 120 consecutive, ceramic THAs in patients under 55 with 96.5% 10-year implant survivorship and another report by Murphy and Murphy [47] of 220 alumina ceramic THAs in patients under 50 with 94.9% 15-year survivorship.

**Table 9:** Literature comparison of survivorship between treatment options for younger patients

Study	Procedure	Prosthesis	Date range	Diagnosis (years)	Patient cohort		Avg FU (years)	Survivorship	
					Hips	Female (%)		FU	Rate (%)
Jameson et al. [51]	HRA	Birmingham	2004–2007	<55	254	39	2.3	3.8	97
Matharu et al. [45]	HRA	Birmingham	1997–2006	<50	447	28	10.1	10	96
Woon et al. [52]	HRA	Conserve Plus	1996–2010	<30	53	39	8.2	8	95
Amstutz et al. [53]	HRA	Conserve Plus	1998–2007	<50	350	25	5.5	5	97.8
Krantz et al. [44]	HRA	Conserve Plus and Durom	2007–2008	<30	24	32	4.2	4.9	100
Haddad et al. [23]	HRA	Birmingham	1999–2002	<55	40	25	12	10	100
Matharu et al. [54]	HRA	Conserve Plus and Corin Cormet 2000	2001–2007	<25 + osteonecrosis	20	50	5.2	8.6	100
Matharu et al. [54]	THA	Stryker Accolade II stem and Trident cup	2001–2007	<25 + osteonecrosis	20	38	5.2	7.3	93
Wroblewski et al. [55]	THA	Charnley	1962–1990	<51	1434	61	15	17	83
Current study	HRA	Corin Surface and Biomet ReCap™	2008–2013	<50	1285	26	3.4	12	96

Outcomes for resurfacing are mixed, with registries typically showing lower implant survivorship than published series from dedicated resurfacing surgeons. Hip resurfacing requires a significant learning curve compared to THA [48] and is seldom taught in residency programs.

The most common failure mode for either group was loosening of the femoral component (1.0% in group 1 and 0.5% in group 2). Converting to exclusively uncemented implants eliminated this failure mode. The second most common cause for revision was failure of acetabular ingrowth (0.8% in group 1 and 0.5% in group 2). After introduction of the acetabular component with Magnum™ Tri-Spike supplemental fixation in high-risk cases in 2007, this failure mode reduced from 1.1 to 0.5%. Since 2010, there have been no instances of failure of acetabular ingrowth in patients under 50 years old.

AWRF is a well-publicized and widely feared complication of MOM HRA. Particle-driven tissue inflammation has long been associated with polyethylene, polymethyl methacrylate, and metallic debris between modular junctions [28]. Tissue inflammation due to metallic particles from excessive MOM bearing wear came to widespread attention around 2007 [34]. At first, many speculated that these represented allergic reactions to metal [22]. Later, studies revealed the cause of the problem as edge-loading on the malpositioned acetabular component [35]. When this occurs, the fluid film lubrication of the hip joint breaks down, and a high wear rate ensues. Metal particles settle in surrounding tissue, activating an inflammatory response. Poor implant design and improper acetabular component positioning cause this abnormal edge-loading wear; thus, smaller bearing sizes, used primarily in women, are more prone to edge-loading because of their lower coverage arc. In response to these findings, we published the RAIL guideline [33], which indicates safe positions for all implant sizes. Patients under 50 presented significantly reduced ion levels when their implant was placed under the RAIL limit, with a mean Co level of 1.4 compared to 2.1 in patients over RAIL (*p* < 0.0001). No instances of AWRF, problematic ion levels, or potentially toxic ion levels occurred in cases under RAIL, which has been achieved in all cases since 2009. These results justify that AWRF is completely preventable in MOM implants, even in cases previously considered high-risk.

This study contains a few notable limitations. First, all HRAs reported herein were performed by a single, experienced HRA surgeon. Registry results are inferior, but numerous publications from experienced HRA surgeons show similar survivorship. Although studies have shown proper HRA surgical technique requires an extended learning curve [27, 48, 49], this curve is expected to shorten with increased availability of HRA research. The next limitation derives from excellent patient outcomes, which recently have been so good that our current clinical measurement, the HHS, suffers from a ceiling effect [50]; a younger patient who outperforms an older patient with a clinical HHS of 100 would still score the same, limiting comparison. Finally, a larger proportion of group 2 received the superior uncemented Biomet ReCap™ device (*p* < 0.0001), potentially skewing results in favor of the older patient cohort. Despite this, postoperative results for the younger study group were similar to, and in many instances better than, those for group 2.

## Conclusions

We make the following conclusions:

- In the largest (*n* = 1285) single series of hip arthroplasty ever published for patients under 50, MOM HRA exceeds the 2014 NICE implant survivorship criteria, with 96.5% at 10 years and 96.4% at 12 years.
- Results in women have improved rapidly, reducing the disparity in outcomes between sexes; furthermore, after eliminating the initial Corin cohort, 10-year implant survivorship for men and women Biomet implants both exceed the 2014 NICE criteria independently at 99 and 97%, respectively.
- There is no difference in survivorship by age among our patients (96.5 vs. 96.3% at 12 years for under and over 50, respectively).
- AWRF is a rare complication (0.3%). Blood ion testing is an effective screening tool. When the acetabular component position meets RAIL guidelines, this failure mode is completely avoidable. None have occurred in 1530 consecutive cases since July 2009.

## Abbreviations

AWRF: Adverse wear-related failure
Co: Cobalt
Cr: Chromium
HHS: Harris hip score
HRA: Hip resurfacing arthroplasty
KM: Kaplan-Meier
MOM: Metal-on-metal
NICE: National Institute for Health and Care Excellence
RAIL: Relative acetabular inclination limit
ROM: Range of motion
THA: Total hip arthroplasty
UCLA: University of California, Los Angeles
VAS: Visual analog scale
